# Adjunctive rifampin for the treatment of *Staphylococcus aureus* bacteremia with deep infections: A meta-analysis

**DOI:** 10.1371/journal.pone.0230383

**Published:** 2020-03-19

**Authors:** Huan Ma, Jie Cheng, Lengyue Peng, Yawen Gao, Guangli Zhang, Zhengxiu Luo

**Affiliations:** 1 Department of Respiratory Medicine, Children's Hospital of Chongqing Medical University, Chongqing, China; 2 Chongqing Key Laboratory of Pediatrics, Chongqing, China; 3 Department of Children's Hospital of Chongqing Medical University of Education, Key Laboratory of Child Development and Disorders, Chongqing, China; Azienda Ospedaliera Universitaria di Perugia, ITALY

## Abstract

**Background:**

*Staphylococcus aureus* (*S*. *aureus*) bacteremia (SAB) has high morbidity and mortality, with the development of methicillin-resistant *S*. *aureus* (MRSA) and the recognized shortcomings of vancomycin, its management is becoming more complicated. Considering the capability to penetrate cells, tissues and biofilms, rifampin has been used as adjunctive agent to against staphylococcal activity.

**Objectives:**

We performed this meta-analysis, aimed to explore the efficacy of adjunctive rifampin for the treatment of SAB.

**Methods:**

Medical literatures were searched in the Pubmed, Medline, Embase and Cochrane databases up to October 2018. Patients with SAB received treatment with or without rifampin were included. The risk ratio (RR) and 95% confidence intervals (CI) of mortality, rate of bacteriological failure and relapse were estimated.

**Results:**

Seven articles (five randomized controlled trials and two retrospective cohort studies) enrolling 979 and 636 patients of SAB treated with and without rifampin, respectively, were included. There was no difference of mortality between the adjunctive rifampin therapy and standard therapy on SAB (RR: 0.771, 95% CI: 0.442 to 1.347, I^2^ = 70.4%). In the subgroup analyses, the decreased mortality was observed in the adjunctive rifampin treatment for patients without MRSA infection (RR: 0.509, 95% CI: 0.372 to 0.697, I^2^ = 8.8%). In addition, there was no difference of the rate of bacteriologic failure (RR: 0.602, 95% CI: 0.198 to 1.825, I^2^ = 0.0%) or relapse (RR: 0.574, 95% CI: 0.106 to 3.112, I^2^ = 77.9%) between the adjunctive rifampin therapy and standard therapy on SAB.

**Conclusions:**

In general, insufficient evidence supported the efficacy of adjunctive use of rifampin for treatment of SAB, adding rifampin to standard therapy didn’t decrease the incidence of death, rate of bacteriologic failure and relapse.

## Introduction

*Staphylococcus aureus* (*S*. *aureus*) is known as an important human pathogen of serious bacterial infections, frequently leads to bacteremia, pneumonia [1], further causes metastatic infections such as infective endocarditis (IE) [2], osteomyelitis [3], skin and soft-tissue infection [1] through the blood migration. Among these infections, *S*. *aureus* bacteremia (SAB) has been recognized with significant morbidity and mortality [4,5]. Although anti-staphylococcal β-lactamase-resistant penicillin has been recommended as the mainstay of methicillin-sensitive *S*. *aureus* (MSSA) management, with the development of methicillin-resistant *S*. *aureus* (MRSA), the management of SAB is becoming even more complicated [6,7]. As the effective therapeutic option for most gram-positive organisms through inhibition of cell wall synthesis, vancomycin has been recommended as the treatment of most infections of MRSA [8,9]. Nevertheless, when considering of its shortcomings such as the increasing minimum inhibitory concentrations (MICs), slow bactericidal activity, poor tissue penetration, reduced activity against biofilm pathogens [10], some other antibiotics with anti-MRSA activity, including linezolid, daptomycin, gentamicin and rifampin, have been suggested as alternatives [11,12]. Among those alternatives, with the capability to penetrate cells and various tissues [13,14], rifampin was regarded as an effective agent to improve antibacterial action and broaden the spectrum of anti-staphylococcal activities [12]. However, the doubt on the use of rifampin in all infections due to *S*. *aureus* is well known. Evidence has indicated the benefits of adjunctive rifampin therapy on those *S*. *aureus* infections involving prostheses, such as prosthetic valve endocarditis (PVE) and prosthetic joint infection (PJI) [8,9,15,16], in contrast, adding rifampin for the treatment of *S*. *aureus* infections not involving inserted medical devices, was not recommend [8,9]. Despite that, as shown in a previous survey, infectious diseases consultants (IDCs) were asked to give treatment choices for a patient with persistent MRSA bacteremia, more than half of IDCs still chose to add rifampin when MIC of vancomycin approaching the limit of the susceptible range [17]. Meanwhile, clinical cases that add rifampin to SAB patients (regardless foreign devices presented or not) were not rare, in regard to evaluation of the efficacy of adjunctive rifampin, two systematic reviews have been published so far, one conducted by Russell CD et al. [18] indicated the possible benefits of adjunctive rifampin on reducing mortality and clinical/bacteriological failure, yet another one [19] drew opposite conclusion. Considering the complicated management of SAB and controversial opinion regarding adjunctive use of rifampin, further research is needed. Therefore, we decided to update present available evidence, by enlarging the numbers of trials and enrolling more subjects, information was collected from studies involving patients with SAB. We aimed to better define the efficacy of adjunctive rifampin for the treatment of SAB, with respect to the rate of death, bacteriological failure and relapse.

## Methods

### Information sources and search key words

Using the Pubmed, Medline, Embase and Cochrane databases, searches for relevant articles were performed with the following items: (rifampin [Title/Abstract] OR rifampicin [Title/Abstract]) AND (*Staphylococcus aureus* [Title/Abstract] OR *S*. *aureus* [Title/Abstract]). Searches were limited to articles published in English till October 2018. Furthermore, additional references were identified from citations in the articles that were reviewed.

### Eligible criteria

The related literatures were evaluated by reviewing the titles and abstracts, and further assessed by reviewing the full texts. Studies involving patients with SAB were included. Participants received two types of therapy: standard therapy (may differed among each study) and standard therapy combined with rifampin, otherwise the treatments were regarded as ineligible. We selected the incidence of death as primary outcome, rate of bacteriologic failure and relapse as secondary outcomes, studies recorded other unsuitable outcomes were not included. Case reports, reviews, notes or comments were excluded.

### Data extraction and quality assessment

Information extracted included first author name, year of publication, study design, pathogen, sites of deep infection, daily rifampin dose, duration of rifampin therapy, standard therapy, number of subjects in each group, number of event and nonevent in each group, primary and secondary outcomes. The Cochrane tool for assessing the risk of bias was used to assess the quality of each RCT [20]. The risk of bias was assessed according to seven criteria including random sequence generation, allocation concealment, blinding of participants and personnel, blinding of outcome assessment, incomplete outcome data, selective reporting, and other bias. Each item was judged to obtain an assessment of ‘low risk’, ‘high risk’ or ‘unclear risk’. The quality of each cohort study was assessed according to Newcastle-Ottawa Scale (NOS) [21], the total score ranges from 0 to 9, and a higher score indicates higher quality. Disagreements among the investigators were resolved by review of the assessments to reach consensus. Quality of evidence (QoE) for outcomes reported in the included trials was assessed using the GRADE methodology and GRADE pro (Computer program located at https://www.gradeworkinggroup.org/). The GRADE system offers four categories of the quality of the evidence (i.e. high, moderate, low and very low).

### Statistical analysis

Statistical analyses were performed using the Stata Version 15.0 software. Pooled risk ratio (RR) and 95% confidence intervals (CI) for all the outcomes were evaluated, considering the variations of the included studies, random effects model was used in all analyses. The statistical heterogeneity was determined by chi-square test and I^2^ statistics (P value ≤ 0.10 and/or I^2^>50% was considered to be significant). To explore the possible sources of heterogeneity, sensitive analysis and subgroup analysis were conducted, subgroups were stratified according to the study designs (cohort study and RCT) and situation of MRSA infection (presence or absence of MRSA infection). Publication bias was visually evaluated using funnel plots and statistically assessed using Egger’s and Begg’s tests. Statistical significance was set at a P value < 0.05 for all analyses.

## Results

### Searching results

**Fig 1:**
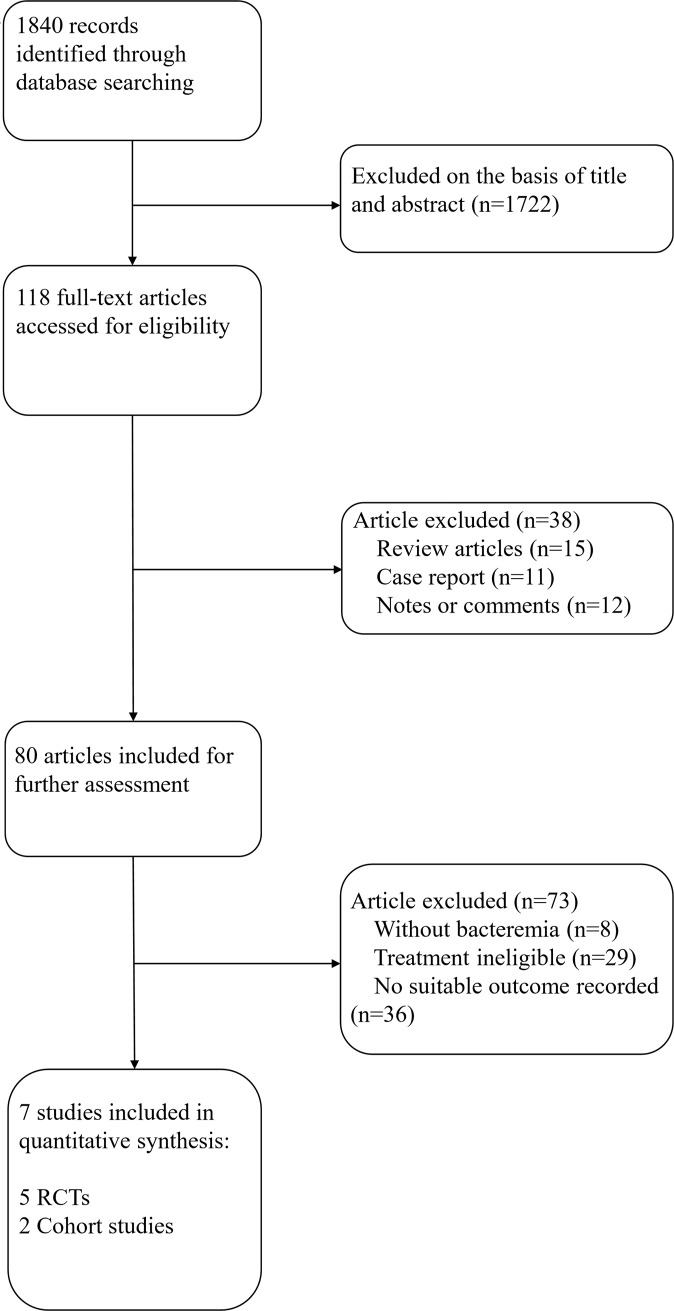
Flowchart and study selection.

Searches of literatures initially identified 1840 potentially relevant records. Following review of the title and abstract, 1722 records were excluded, and a further 111 were excluded following full-text review to give 7 eligible studies, as shown in Fig 1. The reasons for further exclusion included: review articles (n = 15), case report (n = 11), notes of comments (n = 12), no bacteremia (n = 8), treatment ineligible (n = 29) and no suitable outcomes recorded (n = 36).

### Characteristics of the studies

A total of 7 studies [22–28] (five randomized controlled trials and two retrospective cohort studies) published from 1983 to 2017 containing 979 and 636 patients treated with or without rifampin, respectively, were included in the meta-analysis. *S*. *aureus* was the only isolated pathogen in studies, information of MRSA was given in five studies but missed in two [22,23]. Deep infections varied among each study, containing pneumonia, endocarditis, osteomyelitis and foreign body infections and so on. Details regarding daily dose and duration of rifampin therapy were given in six studies (ranged from 450 to 1200mg via intravenously or orally) but missed in one [26]. Standard therapy also varied, vancomycin was the commonest used agent, others including oxacillin, penicillin, cefuroxime, daptomycin, and so on. Primary outcome was reported in all studies, rate of bacteriologic was reported in three studies [22,23,28], and three studies [26–28] provided data of relapse rate. The information of the included articles is summarized in Table 1.

**Table 1:** Characteristics of included studies.

1st author (year of publication)	Design	Pathogen	Deep infections	Rifampin therapy		Group (n)	Outcome measures		
		(ratios of MRSA to *S*. *aureus*)		Daily dose	Duration		Mortality (n)	Bacteriologic failure (n)	Relapse (n)
Van der Auwera P (1983) [22]	RCT	S. aureus (NG#)	Pneumonia, urinary tract infection, osteomyelitis, postoperative wound infection, endocarditis	600 mg, IV then PO	3–43 days	Standard+rifampin (10)	0	0	/
						Standard (9)‖	4	1	/
Van der Auwera P (1985) [23]	RCT	S. aureus (NG)	Pneumonia, urinary tract infection, osteomyelitis, cellulitis	1200 mg, PO	18.9, 21.1 days*	Standard+rifampin (13)	1	0	/
						Standard+placebo (16)	0	3	/
Levine DP (1991) [24]	RCT	S. aureus (100%)	Endocarditis	600 mg, PO	28 days	Standard+rifampin (18)	1	/	/
						Standard (19)	2	/	/
Ruotsalainen E (2006) [25]	RCT	S. aureus (0%)	Endocarditis, pneumonia, deep-seated abscess, osteomyelitis, septic arthritis	450/600 mg, PO or IV§	>28 days	Standard+rifampin (265)	44	/	/
						Standard (66)	25	/	/
Riedel DJ (2008) [26]	Cohort	S. aureus (76%)	Endocarditis	NG	14–48 days	Standard+rifampin (42)	9	/	9
						Standard (42)	2	/	14
Forsblom E (2015) [27]	Cohort	S. aureus (0%)	Pneumonia, endocarditis, purulent arthritis, osteomyelitis, deep-seated abscess and any foreign-body infection	450/600 mg, IV§	Short term: 1–13 days, Long term: ≥14 days	Standard+rifampin (261)	41	/	2
						Standard (96)	25	/	2
Thwaites GE (2017) [28]	RCT	S. aureus (6%)	Endocarditis, prostheses infections, skin or soft tissue infections	600/900 mg, PO or IV§	14 days	Standard+rifampin (370)	56	4	3
						Standard+placebo (388)	56	5	16

Abbreviation and notes:

#, Not given: information was not given

§, [25,27] Rifampicin was administered 450 mg once daily for patients under 50 kg and 600 mg once daily for patients over 50 kg in weight, [28] 600 mg or 900 mg of rifampicin was given per day according to weight

*, mean duration of therapy

‖,Standard therapy: [22,23] oxacillin, vancomycin, [24] vancomycin, gentamicin, [25] semisynthetic penicillin, levofloxacin, cloxacillin, cefuroxime, clindamycin, vancomycin, [26] vancomycin, nafcillin, daptomycin, cefazolin, [27] cloxacillin, cefuroxime, ceftriaxone, vancomycin, clindamycin, fluoroquinolone, aminoglycoside, [28] flucloxacillin, vancomycin, teicoplanin.

### Quality assessment

**Fig 2:**
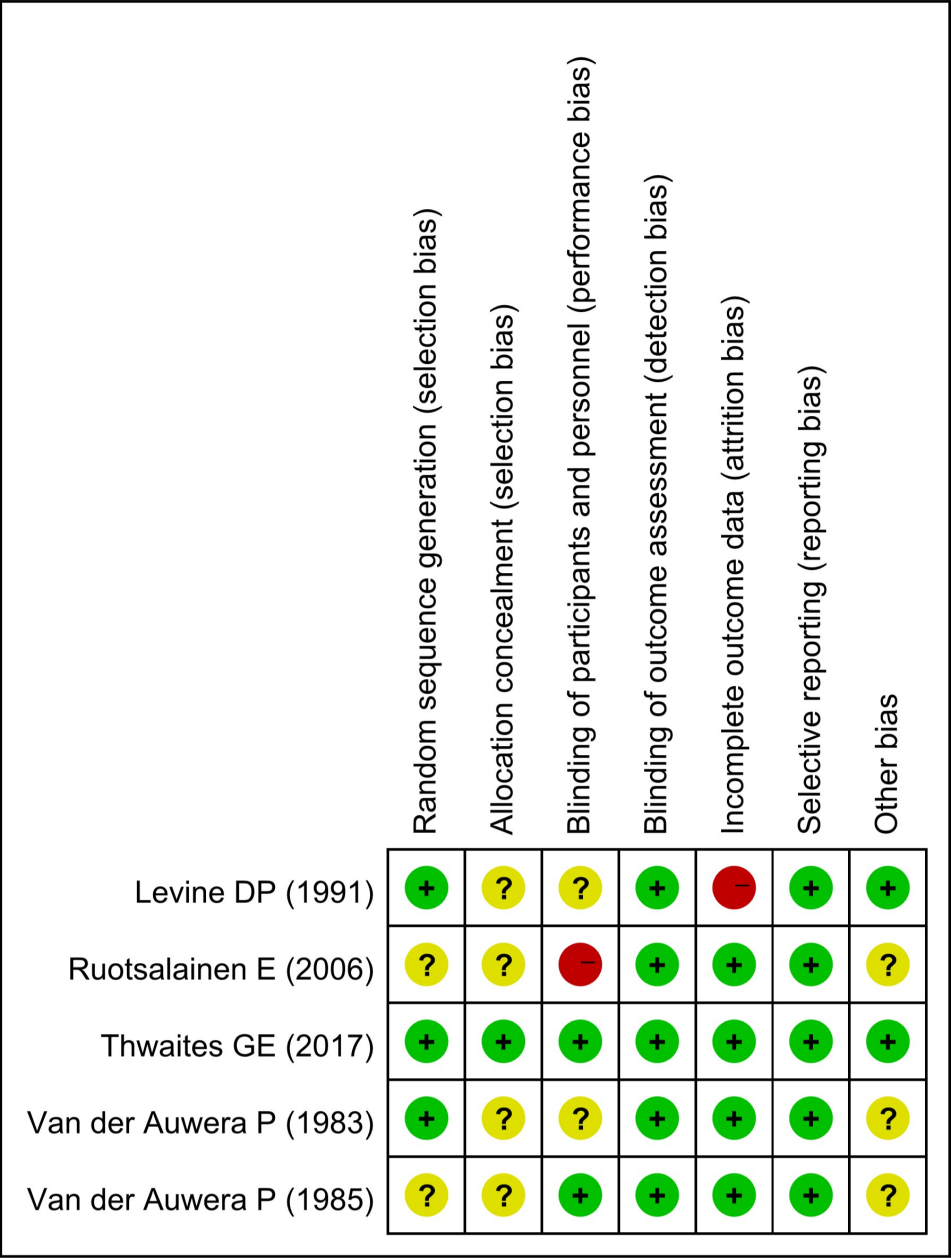
Risk of bias summary for each included RCT.

In general, the scores of two retrospective cohort studies are both eight, the risk of bias of RCTs was unclear or low. The lack of blinding of participants and personnel and incomplete outcome data in two studies led to an increase of the risk of bias to some degree, the result of quality assessment is given in Fig 2. Using the GRADE, the overall QoE for all assessed outcomes was rated as moderate to low (S1 Table).

### Pool analysis

#### Primary outcome: Mortality

**Fig 3:**
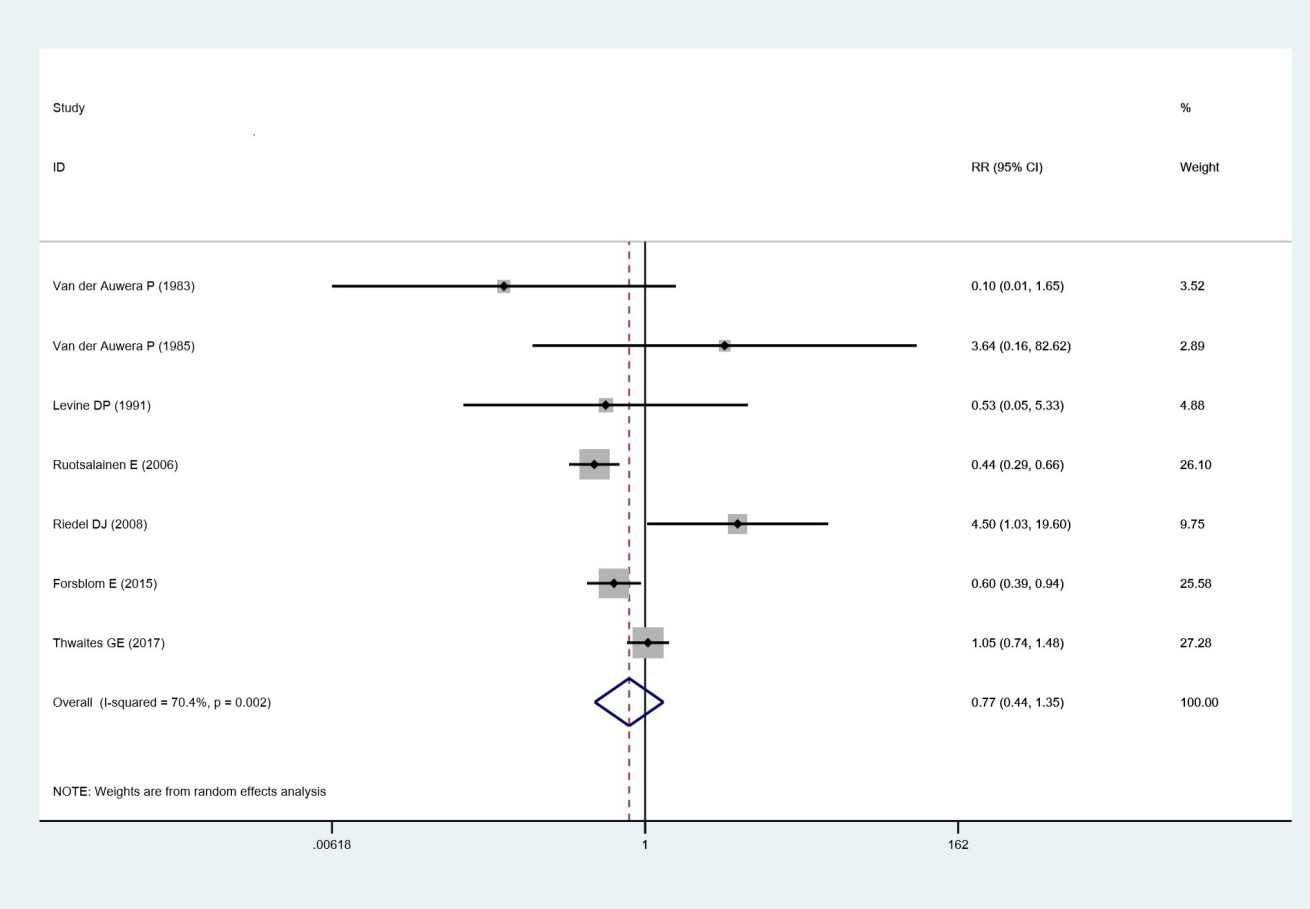
Forest plot: Impact of adjunctive rifampin therapy on mortality of SAB. RR, risk ratio, CI, confidence interval.

Seven studies [22–28] reported the rate of death in SAB patients. Pooled analysis showed there was no difference of mortality between adjunctive rifampin treatment group and control group (RR: 0.771, 95% CI: 0.442 to 1.347, random effects model), a significant heterogeneity was observed (I^2^ = 70.4%, P = 0.002), as shown in Fig 3.

**Fig 4:**
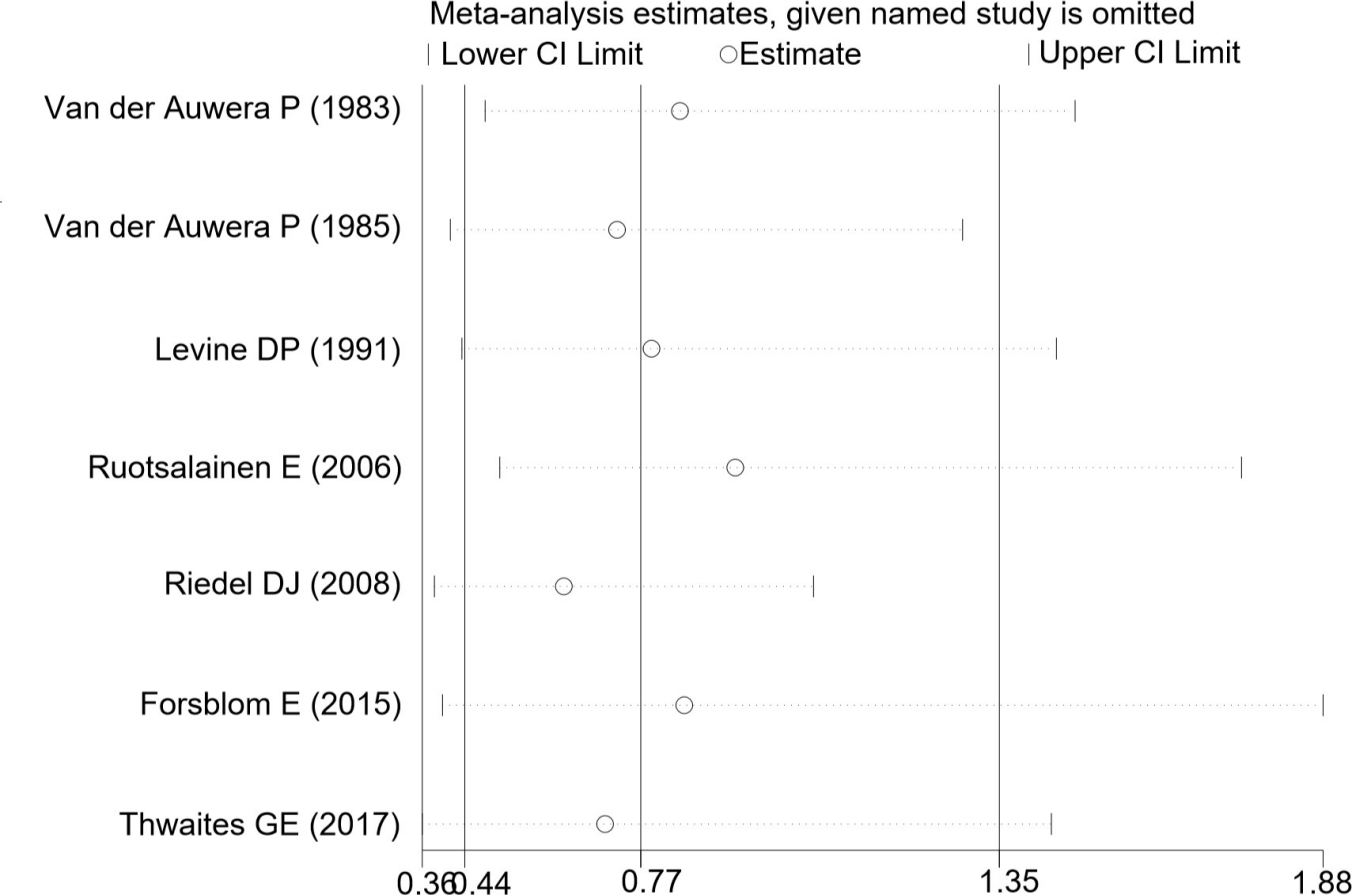
Sensitivity analysis of the included articles. CI, confidence interval.

Sensitivity analysis showed that removal of each single study did not alter the overall results of pooled analyses (Fig 4). High heterogeneity was partly minimized through further subgroup analysis. Adding rifampin for the treatment of patients without MRSA infection, the rate or mortality was reduced compared to control group (RR: 0.509, 95% CI: 0.372 to 0.697), with low heterogeneity was observed (I^2^ = 8.8%, P = 0.295). However, there was no statistical difference of mortality in other subgroups, as shown in Table 2.

**Table 2:** Subgroup analyses: Impact of adjunctive rifampin on SAB mortality.

Subgroups		N	Test for overall effect		Test for Heterogeneity	
			RR (95%CI)	P	P	I^2^
Study design	RCT	5	0.647 (0.307,1.362)	0.252	0.009	70.2%
	Cohort	2	1.459 (0.197,10.830)	0.712	0.009	85.5%
MRSA infection	Yes	3	1.404 (0.509,3.871)	0.512	0.134	50.2%
	No	2	0.509 (0.372,0.697)	0.000*	0.295	8.8%
	NG	2	0.566 (0.017,19.330)	0.752	0.092	64.9%

Abbreviation and notes: NG, information was not given; N, the number of articles; RR, risk ratio; 95% CI, 95% confidence interval

*, significant difference.

#### Secondary outcomes

**Fig 5:**
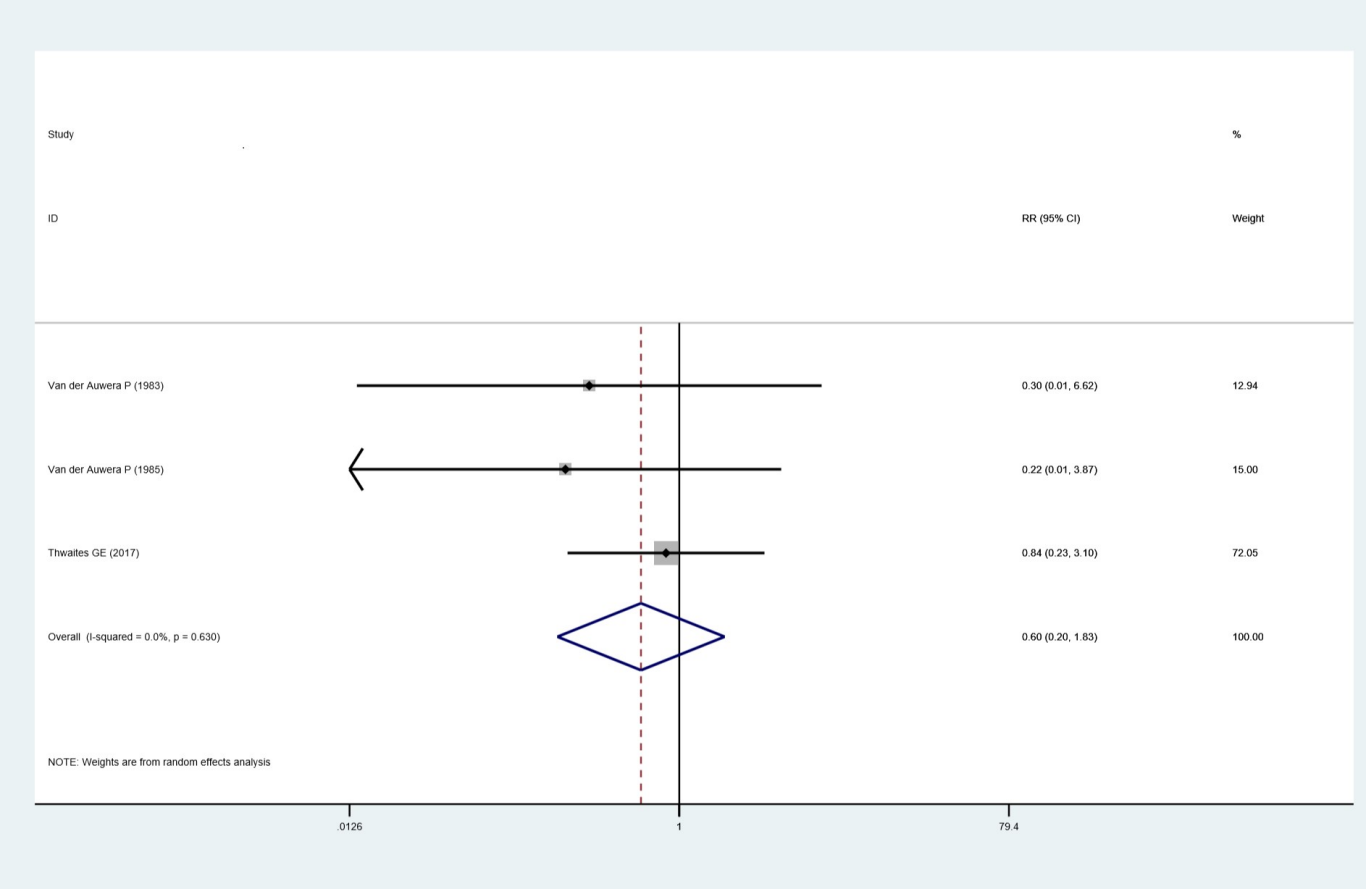
Forest plot: Influence of adjunctive rifampin therapy on rate of bacteriologic failure. RR, risk ratio, CI, confidence interval.

*Rate of bacteriologic failure*. Three studies [22,23,28] reported the rate of bacteriologic failure in SAB patients. There is no difference of bacteriologic failure between adjunctive rifampin treatment and standard therapy (RR: 0.602, 95% CI: 0.198 to 1.825, random effects model), with no heterogeneity was observed (I^2^ = 0.0%, P = 0.630), as shown in Fig 5.

**Fig 6:**
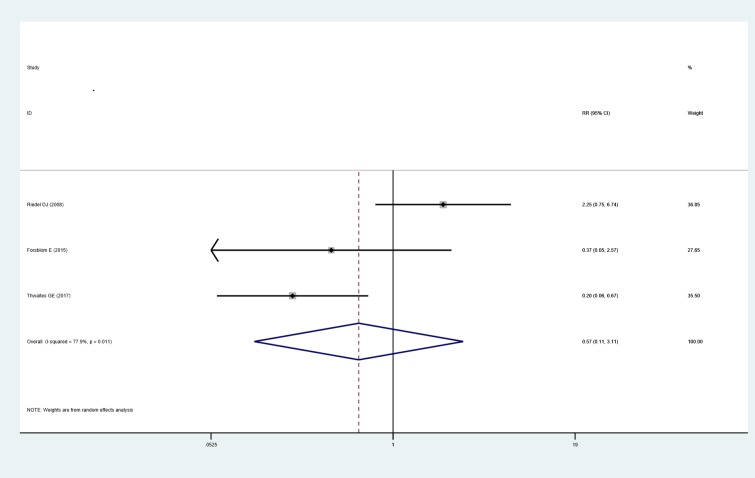
Forest plot: Influence of adjunctive rifampin therapy on relapse rate. RR, risk ratio, CI, confidence interval.

*Rate of relapse*. Three studies [26–28] reported the rate of relapse in SAB patients. There is no difference of relapse rate between patients treated with and without addition of rifampin after pooling the data with meta-analysis (RR: 0.574, 95% CI: 0.106 to 3.112), a significant heterogeneity was observed (I^2^ = 77.9%, P = 0.011), as shown in Fig 6.

### Publication bias

**Fig 7:**
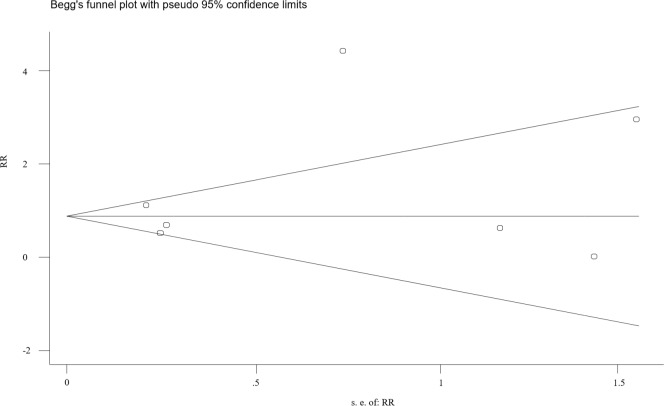
Funnel plots for assessing publication bias of the included studies. SE, standard error, RR, risk ratio.

There appeared to be funnel plot asymmetry for the incidence of death (Fig 7), but Begg’s and Egger’s test indicated no evidence of publication bias (P Begg: 0.548 and P Egger: 0.334).

## Discussion

Rifampin is known as an effective antibacterial agent with the ability of penetrating cells and various tissues, againsting intracellular phagocytized *S*. *aureus* and preventing the haematogenous dissemination [29,30]. Therefore, it was adjunctively used for the treatment of *S*. *aureus* infection, aimed to improve antibacterial action and broaden the spectrum of anti-staphylococcal activity. So far, two systematic reviews evaluating efficacy of adjunctive rifampin therapy for SAB, have been published: one indicated the reduced mortality, clinical or bacteriological failure in adjunctive rifampin group [18], yet another one [19] demonstrated adding rifampin showed no benefits on decreasing survival rates, even further indicated the prevalence of rifampin resistance and prolonged duration of bacteremia. Given the above inconsistent opinion and out of date studies included in both reviews, we aimed to update present evidence by enlarging the accessible studies with our best efforts.

Pooled analyses of our meta analyses showed there is no difference of incidence of mortality, rate of bacteriologic failure or relapse between adjunctive rifampin group and control group. Several explanations exist for the results.

First one should be the development of rifampin resistance, mechanism of which is known as the single bp changes in the b-subunit of the rpoB encoded RNA polymerase [31]. Previous studies already indicated the correlation of rifampin resistance development and decreased microbiological eradication rate [32,33]. Although the resistance was considered most likely to developed when rifampin was used as monotherapy, relevant reports were also not rare in combination therapy [32–36]. Of the included studies, one study [26] demonstrated 9 of 42 patients who received rifampin combination therapy developed rifampin-resistance, longer duration of bacteremia and were less likely to survive than controls. Another one [28] reported rifampin resistance was developed in 2 of 56 patients. However, both studies didn’t further compare the microbiological eradication rate between patients with and without rifampin resistance. In addition, the commonest used standard therapy in the included studies was vancomycin, yet concentration of which was showed varying greatly among organs including heart valve, lung, breast, subcutaneous fat, and muscle tissue [37,38], inversely, rifampin has sustained penetration into the above and other organs [39,40]. Of patients recruited in our meta analyses, most had infection of various organs such as lung, endocardium and so on, therefore, in the case of tissues solely exposed to rifampin, the rapid development of rifampin resistance may be easier developed.

Furthermore, other explanation may be the antagonism effect of rifampin and standard antibiotics when used as clinical combination. Vitro studies investigating the anti-staphylococcal activity of rifampin combination therapy often yielded conflict results, both antagonism and synergy effect were observed [41–46]. This phenomenon may due to the different test system utilized, for instance, different result might be obtained from timed-kill and checkerboard method [47]. Whether two agents show antagonism or synergy effect while combined using may also be affected by the serum concentration: the reduced killing activity was observed when the concentration ratio of standard antibiotic to rifampin was high, but was enhanced at low ratio [45]. This explanation can be illustrated by the results of two included studies: when rifampin was added during the early treatment period which the serum concentration of standard antibiotic was relatively high, a reduction in bactericidal activity was observed [22]; similarly, in another study, patients who failed therapy tended to have higher serum concentration of standard antibiotic than those with a satisfactory therapy response [24].

Moreover, as the potent inducer of the cytochrome p450 system, rifampin is well documented to cause clinically significant drug interactions, including interactions with cardiovascular drugs, antidiabetic agents, antibacterials such as linezolid, moxifloxacin, and so on [48]. A previous study reported a possible interaction between linezolid and rifampin in the combination therapy of MRSA infection, which further leading to the decreased serum levels of linezolid [49]. Nevertheless, none of the included studies evaluating the drug interaction between rifampin and standard antibiotics, whether it existed or how much it contributed to the treatment outcome is uncertain.

Additionally, we tried to explore the possible sources of heterogeneity, subgroups first were stratified according to different study design (cohort study or RCT), in each subgroup, no statistical difference of mortality was detected and the high heterogeneity cannot be minimized. We further conducted subgroup analysis according to the situation of MRSA infection (presence or absence of MRSA infection). Reduced mortality rate of adjunctive rifampin group was only detected in patients without MRSA infection (MSSA bacteremia). However, before we consider the possible benefits of adjunctive rifampin on MSSA bacteremia, a fact shouldn’t be ignored: compared to patients who receive rifampin, the patients treated without rifampin were significantly older, significantly more often had chronic renal failure, a fatal underlying disease or hospital-acquired SAB [25], or had a higher rate of healthcare associated SAB, which might lead to poorer SAB outcome [27].

Meanwhile, two included studies reported other events during treatment: Forsblom E et al. [27] compared the incidence of severe bacteremia and septic shock between short term rifampin therapy (0–13 days) and standard therapy, no difference was observed. In another study [24], one and zero patient developed into septic shock in rifampin group and control group, respectively. Whereas there were insufficient data to define the overall difference regarding those outcomes.

Our meta-analysis was performed based on a highly sensitive and comprehensive search of the literatures, we aimed to update present evidence by identifying as many relevant studies as possible. However, it has some unavoidable limitations. The first one is the small number of included articles. Despite our best efforts to retrieve all related data, with limited well-structured clinical trials to examine the efficacy of rifampin for SAB, fewer literatures were able to meet our stringent inclusion criteria. The high heterogeneity of pooled analysis would be another limit. Although it was partly minimized through subgroup analysis, other factors such as various sites of infection (the presence or absence of medical devices), variation in standard therapy, variation in dose and duration of rifampin treatment, may co-contribute to the high heterogeneity, yet the absence of the related data limited a subgroup analysis to explore further.

## Conclusion

Based on our results, insufficient evidence supported the efficacy of adjunctive use of rifampin for treatment of SAB, adding rifampin to standard therapy didn’t decrease the incidence of death, rate of bacteriologic failure and relapse.

## Supporting information

S1 Table GRADE evidence profile summarizing the effect of adjunctive rifampin therapy vs. standard therapy on Staphylococcus aureus bacteremia (SAB). (PDF)

S1 File PRISMA checklist. (DOC)
