# Supplementary material for: Adjunctive rifampin for the treatment of Staphylococcus aureus bacteremia with deep infections: A meta-analysis
Source: PLoS One. 2020 Mar 19;15(3):e0230383. doi: 10.1371/journal.pone.0230383 (PMC7082046; doi:10.1371/journal.pone.0230383)
Supplement: S1 Table — (PDF) [file pone.0230383.s001.pdf]

Table S1. GRADE evidence profile summarizing the effect of adjunctive rifampin therapy vs. standard therapy on *Staphylococcus aureus* bacteremia (SAB).

Author(s): Huan Ma
Date: 2020-02-01
Question: Should adjunctive rifampin therapy be used for SAB

Certainty assessment							№ of patients		Effect		Certainty	Importance
№ of studies	Study design	Risk of bias	Inconsistency	Indirectness	Imprecision	Other considerations	Adjunctive rifampin therapy	placebo	Relative (95% CI)	Absolute (95% CI)		
**Evidence from RCTs: comparing the incidence of death between adjunctive rifampin therapy and standard therapy.**												
5	randomised trials	serious [a]	serious [b]	not serious	not serious	none	102/676 (15.1%)	87/498 (17.5%)	**RR 0.65** (0.31 to 1.36)	**61 fewer per 1,000** (from 121 fewer to 63 more)	⨁⨁◯◯ LOW	CRITICAL
**Evidence from cohort studies: comparing the incidence of death between adjunctive rifampin therapy and standard therapy.**												
2	observational studies	not serious	serious [b]	not serious	serious [c]	none	50/303 (16.5%)	27/138 (19.6%)	**RR 1.46** (0.20 to 10.83)	**90 more per 1,000** (from 157 fewer to 1,000 more)	⨁⨁◯◯ LOW	CRITICAL
**Evidence from RCTs: comparing the rate of bacteriologic failure between adjunctive rifampin therapy and standard therapy.**												
3	randomised trials	serious [d]	not serious	not serious	not serious	none	4/390 (1.0%)	9/413 (2.2%)	**RR 0.60** (0.20 to 1.83)	**9 fewer per 1,000** (from 17 fewer to 18 more)	⨁⨁⨁◯ MODERATE	IMPORTANT

CI: Confidence interval; **RR:** Risk ratio

Explanations

a. Of the 5 included trials, in 2 there was unclear random sequence generation, in 4 there was an unclear risk of bias for allocation concealment, in 1 there was no blinding of participants and personnel, and in 2 trials blinding was unclear. The blinding of outcome assessment was unclear in 8 trials and no blinding in 1 trial. Incomplete outcome assessment was high risk in 1 trial, there was unclear other bias in 3 trials.
b. Statistical heterogeneity between the included studies
c. there were only 2 studies and total number of events < 500
d. Of the 3 included trials, in 1 there was unclear random sequence generation, in 2 there was an unclear risk of bias for allocation concealment, in 1 trial, there was unclear blinding of participants and personnel, there was unclear other bias in 2 trials.
